# Lower Extremity Microembolism in Open vs. Endovascular Abdominal Aortic Aneurysm Repair

**DOI:** 10.3389/fsurg.2016.00018

**Published:** 2016-03-31

**Authors:** Shahab Toursavadkohi, Stavros K. Kakkos, Ilan Rubinfeld, Alexander Shepard

**Affiliations:** ^1^Trauma Surgery, Henry Ford Hospital, Detroit, MI, USA; ^2^Vascular Surgery, University Hospital of Patras, Patras, Greece; ^3^Division of Vascular Surgery, Department of Surgery, Henry Ford Hospital, Detroit, MI, USA

**Keywords:** open AAA repair, EVAR, microemboli, trash foot, abies, toe pressure

## Abstract

Although previous studies have documented the occurrence of microembolization during abdominal aortic aneurysm (AAA) repair by both open and endovascular approaches, no study has compared the downstream effects of these two repair techniques on lower extremity hemodynamics. In this prospective cohort study, 20 patients were treated with endovascular aneurysm repair (EVAR) (11 Zenith, 8 Excluder, and 1 Medtronic) and 18 patients with open repair (OR) (16 bifurcated grafts, 2 tube grafts). Pre- and postoperative ankle-brachial indices (ABIs) and toe-brachial indices (TBIs) were measured preoperatively and on postoperative day (POD) 1 and 5. Demographics and preoperative ABIs/TBIs were identical in EVAR (0.97/0.63) and OR (0.96/0.63) patients (*p* = 0.21). There was a significant decrease in ABIs/TBIs following both EVAR (0.83/0.52, *p* = 0.01) and OR (0.73/0.39, *p* = 0.003) on POD #1, although this decrease was greater following OR than EVAR (*p* = 0.002). This difference largely resolved by POD #5 (*p* = 0.41). In the OR group, TBIs in the limb in which flow was restored first was significantly reduced compared to the contralateral limb (0.50 vs. 0.61, *p* = 0.03). In the EVAR group, there was also a difference in TBIs between the main body insertion side and the contralateral side (0.50 vs. 0.59, *p* = 0.02). Deterioration of lower extremity perfusion pressures occurs commonly after AAA repair regardless of repair technique. Toe perfusion is worse in the limb opened first during OR and on the main body insertion side following EVAR, suggesting that microembolization plays a major role in this deterioration. The derangement following OR is more profound than after EVAR on POD #1, but recovers rapidly. This finding suggests that microembolizarion may be worse with OR or alternatively that other factors associated with OR (e.g., the hemodynamic response to surgery with redistribution of flow to vital organs peri-operatively) may play a role.

## Introduction

Abdominal aortic aneurysm (AAA) is a degenerative process frequently associated with significant thrombus and debris within the aneurysm sac. The relationship between such disease and distal arterial embolism has been well recognized since its first description by Flory in 1945 (1). Microembolization of such debris can be associated with significant morbidity and mortality depending on the amount of embolic material dislodged and the vascular beds affected (2–5).

Since its introduction in the early 1990s, endovascular repair has evolved into an effective alternative to traditional open repair (OR) for patients with AAA. Endovascular AAA repair (EVAR) is associated with lower 30-day operative mortality, decreased blood loss, and shortened hospital stay compared to conventional OR (6, 7). However, EVAR is not without risks, including vascular injury, endoleak, device malfunction, and atheroembolic events (8, 9). Lower extremity cholesterol embolism, or “trash foot,” is a well-documented complication of AAA repair, reported after both OR and EVAR (10).

Thompson and colleagues performed femoral artery ultrasound during both EVAR and OR and showed a greater occurrence of embolism with EVAR (11). In addition, computerized tomography (CT) angiography has documented renal embolic perfusion defects in up to 18% of patients following EVAR (12). The findings of these studies suggest that embolism of aneurysm debris during AAA repair occurs routinely regardless of technique used. To date, however, no study has addressed the effect of this embolism on the macrocirculation of the lower extremities, the vascular bed at most risk. The goal of the current study was to measure this effect following AAA repair comparing EVAR and OR using non-invasive studies of limb perfusion – ankle-brachial and toe-brachial pressure indices.

## Materials and Methods

This study was approved by the Institutional Review Board of the Henry Ford Hospital.

All participants gave written informed consent.

### Subjects

Between April 2008 and May 2009, 43 patients without overt evidence of lower extremity arterial occlusive disease scheduled for elective infrarenal AAA repair were studied prospectively. All patients had an AAA larger than 5 cm in maximum diameter or an increase in aneurysm diameter more than 1 cm over the previous year. Exclusion criteria included: urgent or emergent AAA operation, re-do aortic surgery, anticipated aortic clamping proximal to a renal artery, prior lower extremity amputation, or evidence of concomitant peripheral arterial occlusive disease as manifest by a history of claudication, previous lower extremity bypass surgery, or absence of easily palpable pedal pulses. All patients were evaluated with contrast enhanced computed tomography (CT) preoperatively to define aneurysm morphology. The operative approach for AAA repair was at the discretion of the operating surgeon, member of the vascular surgery division of Henry Ford Medical Group (HFMG).

Ankle pressures were measured in the dorsalis pedis and posterior tibial arteries utilizing a continuous wave Doppler flow probe and a 10 cm blood pressure cuff placed over the lower calf just above the malleoli. An ankle-brachial index (ABI) was obtained by normalizing the higher of the two pressures to the brachial pressure. Toe pressures were determined using a photoplethysmographic probe attached to the tip of the hallux and a small sphygmomanometer cuff on the proximal toe. Toe pressures were also normalized to brachial pressures (toe-brachial index or TBI). This method has previously been validated and described in details by Samuelsson et al. (13). All measurements were performed by the primary investigator, to avoid inter-observer variation. Non-invasive studies were performed immediately preoperatively and on postoperative day (POD) #1 in both groups. A second set of postoperative studies were performed on the OR patients on POD #5. All patients undergoing EVAR were discharged between POD #1 and 3 and did not have a second set of studies performed.

### Statistics

Power calculations determined that 20 patients were needed in each group to demonstrate statistical significance of 0.05 and power of 90%, assuming that a difference of 0.1 in TBI was clinically significant and using previous data on the SD of TBI that is 0.095. Paired and unpaired Student’s *t*-test and Chi-square were used for continuous and categorical variables, respectively, in the two study groups.

Data were expressed as mean ± SD. Data were analysed in Aabel 3.0 (Gigawiz, UK), and *p* < 0.05 was considered significant.

## Results

Of the 43 patients who initially qualified for enrollment into the study, 3 had incomplete data and 2 subsequently withdrew their consent. This left 38 patients with available data for analysis. Patient demographics and associated co-morbidities (hypertension, diabetes, chronic kidney disease, and hypercholesterolemia) were similar in the two groups (Table 1). In the EVAR group, the aneurysm size was larger, 6.1 cm (range 4.9–8.4 cm) vs. 5.6 cm (range 4.8–7.9 cm) (*p* = 0.01) in the OR group, and the infrarenal neck was longer (2.69 vs. 1.68 cm, *p* = 0.01). Aortic clamping was accomplished at an infrarenal site in all patients undergoing OR, and aneurysm reconstruction was performed with a bifurcated graft in 16 cases (88%) and a tube graft in 2 cases (12%). Endovascular repair was performed with the Cook Zenith graft in 11 patients (55%) and the Gore Excluder graft in 9 patients (45%).

**Table 1:** **Baseline characteristics of the patients**.

Characteristic (no.)	EVAR (20)	OR (18)	*p* Value
Age (years)	76 ± 6.1	68 ± 6.3	*p* = 0.14
Male no. (%)	15 (75%)	15 (83%)	*p* = 0.47
Aspirin use no. (%)	12 (60%)	12 (66%)	*p* = 0.52
Statin use no. (%)	13 (65%)	13 (72%)	*p* = 0.31
ACE inhibitor use no. (%)	15 (75%)	12 (66%)	*p* = 0.22
**Co-morbidities (no.)**			
Hypertension no. (%)	18 (90%)	16 (88%)	*p* = 0.57
Diabetes no. (%)	6 (30%)	4 (22%)	*p* = 0.44
Hypercholesterolemia no. (%)	13 (65%)	11 (61%)	*p* = 0.55
Chronic kidney disease (Cr >1.5) no. (%)	7 (35%)	5 (27%)	*p* = 0.23

*EVAR, endovascular aneurysm repair; OR, open abdominal aortic aneurysm repair; ACE, angiotensin inhibitor*.

Duration of operation was significantly longer in the OR group, 6.4 h (range 3.6–10.6 h) vs. 3.4 h (range 2.1–7.5 h) (*p* = 0.02) in the EVAR group (Table 2). No patient in either group developed cutaneous signs of atheroembolization (e.g., blue or discolored toes or livedo reticularis) in the postoperative period.

**Table 2:** **Procedure details**.

	EVAR (20)	OR (18)	*p* Value
Diameter of AAA (cm)	6.1 (4.9–8.8)	5.6 (5.0–7.6)	0.41
Length of aneurysm neck (cm)	2.69 (1.0–4.2)	1.68 (0.9–3.2)	0.01
Type of reconstruction	11 (55%) Zenith 9 (45%) Excluder	16 (88%) bifurcated 2 (12%) tube	
Duration of procedure (h/min)	3:43 (2:05–7:55)	6:42 (3:55–9:35)	0.01

*EVAR, endovascular aneurysm repair; OR, open abdominal aortic aneurysm repair; AAA, abdominal aortic aneurysm*.

**Figure 1:**
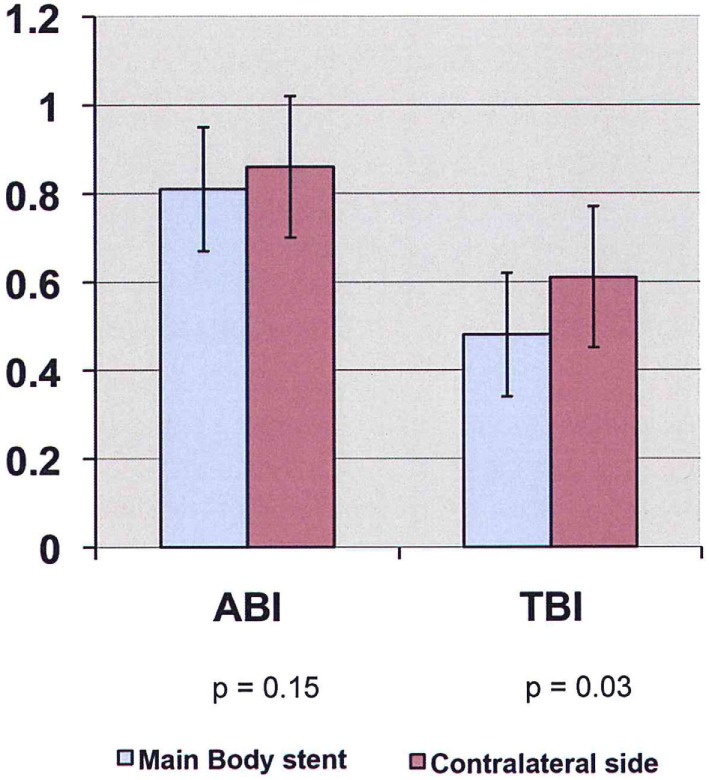
**ABI/TBI changes of limb first perfused vs. contralateral limb in patients undergoing open aneurysm repair on POD #5 (ABI, ankle-brachial index; TBI, toe-brachial index)**.

**Figure 2:**
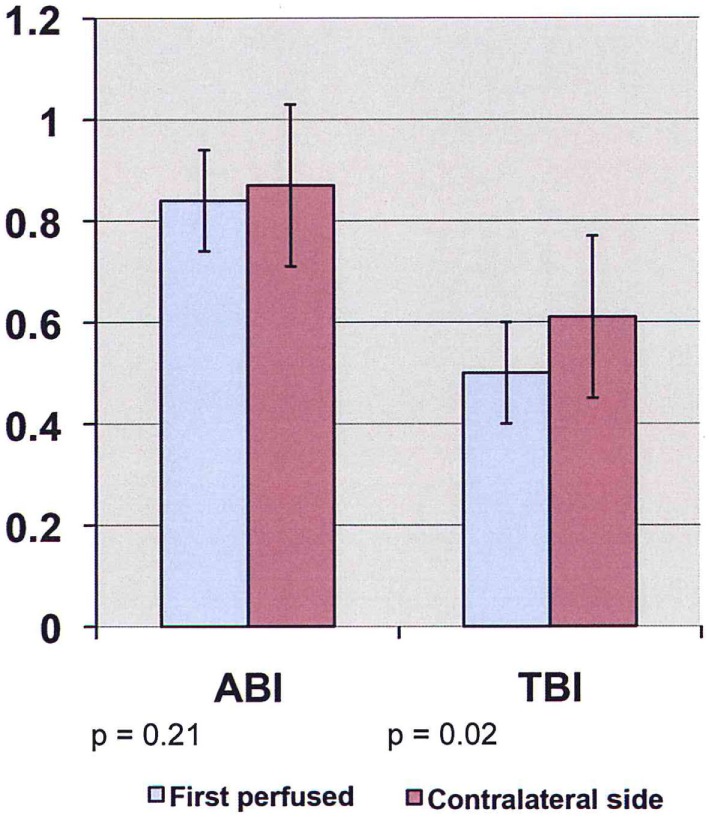
**ABI/TBI changes of main body stent graft limb vs. contralateral limb in patients undergoing endovascular aneurysm repair on POD #1 (ABI, ankle-brachial index; TBI, toe-brachial index)**.

Preoperative ABI/TBI in the two groups were similar 0.97 ± 0.13/0.63 ± 0.07 in the EVAR patients and 0.96 ± 0.18/0.63 ± 0.17 in the OR patients (*p* = 0.21). There was a significant decrease in ABI/TBI on POD #1 following both EVAR 0.83 ± 0.25/0.52 ± 0.15 (*p* = 0.01) and OR 0.73 ± 0.22/0.39 ± 0.19 (*p* = 0.003). This decrease was greater following OR than EVAR (*p* = 0.002) but largely resolved by POD #5: 0.85 ± 0.18/0.53 ± 0.17 (*p* = 0.41). In the OR group, TBI in the limb where flow was restored first was significantly lower than the contralateral limb 0.50 ± 0.12 vs. 0.61 ± 0.18 (*p* = 0.03, Figure 1). In the EVAR group, there was a difference in TBI between the side the main body of the stent graft was inserted and the side the smaller contralateral limb was inserted 0.50 ± 0.14 vs. 0.59 ± 0.11 (*p* = 0.02, Figure 2). A similar difference was not seen in ABIs in either OR – limb first perfused vs. contralateral limb, 0.84 ± 0.16 vs. 0.87 ± 0.19 (*p* = 0.15) or EVAR patients – main body stent graft limb 0.81 ± 0.19 vs. contralateral limb 0.86 ± 0.21 (*p* = 0.21) (Table 3).

**Table 3:** **ABI/TBI changes**.

	Preoperative	POD #1	POD #5
OR	0.96 ± 0.18	0.73 ± 0.22	0.85 ± 0.18
	0.63 ± 0.17	0.39 ± 0.19	0.53 ± 0.17
EVAR	0.97 ± 0.13	0.83 ± 0.25	ND
	0.63 ± 0.07	0.52 ± 0.15	ND

*ABI/TBI, ankle brachial index/toe brachial index; OR, open abdominal aortic aneurysm repair; ND, not done*.

## Discussion

Microembolization of thrombus and/or atherosclerotic debris is a complication that can occur after any vascular operation but is particularly well recognized after AAA repair. The clinical picture is highly variable ranging from asymptomatic toe discoloration to more dramatic organ dysfunction/decompensation including renal failure (2, 3), ischemic colitis (14), multiorgan failure (15, 16), and lower extremity tissue loss (17–19).

Abdominal aortic aneurysm repair is associated with a higher risk of microembolization than other vascular procedures because the aneurysm sac is filled with thrombus and debris, which can be released into the circulation during aneurysm manipulation and proximal and distal clamping and unclamping. The incidence of clinically significant lower extremity embolism following open aortic reconstruction has been reported to range from 0.6 to 5% (18, 19). Embolization of aneurysm debris during EVAR is similarly well-recognized (20). In fact, reports of initial experience with EVAR in the mid-1990s suggested that massive microembolization was a significant complication of this technique occurring in anywhere from 4 to 17% of patients and was associated with mortality (21–23). With refinement of technique and advances in stent graft design the rate of clinically recognized embolization has dropped dramatically and is currently reported to occur in only 0.9% of patients (24). Asymptomatic and/or clinically insignificant microembolization undoubtedly has a much higher incidence.

Previous prospective studies of this problem have been scarce. In the late 1990s, the Leicester Vascular Surgery group used a transcranial Doppler flow probe intraoperatively to monitor the passage of embolic signals in the superficial femoral arteries of patients undergoing AAA repair (11). They compared OR patients with those undergoing EVAR and found that EVAR was associated with a significantly higher number of lower extremity emboli than was OR (120 embolic signals per case in EVAR vs. 31 in OR). These investigators only studied the occurrence of emboli intraoperatively and did not examine or attempt to quantify the impact of these emboli on pedal perfusion or other vascular beds.

The results of the current study document that lower extremity perfusion pressures commonly deteriorate following AAA repair regardless of repair technique. While the cause of this deterioration is undoubtedly multifactorial, it seems likely that microembolization of aneurysm contents plays a major role. This conclusion is supported by the fact that the toe pressure was lower on the side opened first following OR and in the main body insertion side following EVAR. These are the limbs at greatest risk for embolization – during OR the limb opened first accepts any debris released from the aortic and iliac clamp sites as well as the graft itself while the remaining limb is subjected to debris only within the graft limb and at the distal clamp site. Similarly, during EVAR, the main body insertion side would seem more at risk for embolization because of the larger size of the device and introducer sheaths than those used for the contralateral limb.

While one would assume that microembolization is the most important causative factor for the observed drop in toe perfusion pressures, it is impossible to rule out the interplay of other factors. Changes in the patients’ overall hemodynamics with redistribution of flow to vital organs immediately postoperative seems a likely contributing factor in patients undergoing OR and may explain the differences in the two groups seen on POD #1. Degree of resuscitation, patient pain level, and patient temperature are just a few of the factors that could play a role in altering these measurements and it seems likely that all of these factors would be impacted more significantly by OR than EVAR. Alternatively, the greater deterioration in ABI/TBI in OR patients on POD #1 may reflect greater embolization of aneurysm debris during OR. This finding would be in contrast to the study by Thompson who showed more intraoperative lower extremity emboli in patients undergoing EVAR (11). The subsequent recovery in ABI/TBI to near baseline levels by POD #5 suggests that these changes in OR patients are transient.

The fact that clinically significant lower extremity embolic complications after AAA repair are relatively infrequent may be because the lower extremity has a relatively higher tolerance for microembolization than other organs with a more sensitive metabolism (e.g., kidney or brain). Nevertheless, it would appear that microembolization of sac debris is a relatively routine occurrence during AAA repair regardless of repair technique. Although not associated with any clinical sequelae, this embolization does cause some impairment in pedal hemodynamics. The implications of this embolization for other vascular territories affected by AAA repair (i.e., renal and hypogastric arteries and the inferior mesenteric artery) are unclear. Embolic protection devices have the potential to reduce the magnitude of this problem, but may be associated with their own set of complications.

This study has several limitations: patients were not matched for atherosclerotic burden (i.e., severity of generalized atherosclerosis). Patients undergoing EVAR were not followed up after POD #1 and patients were not followed until ABI/TBIs normalized. Knowledge of the speed of recovery would be informative, but the amount of work required was beyond the scope of this project.

## Author Contributions

ST obtained the IRB and performed the research. AS was the PI with the original concept. SK assisted the design of this study. IR performed all statistical analysis.

## Conflict of Interest Statement

The authors declare that the research was conducted in the absence of any commercial or financial relationships that could be construed as a potential conflict of interest.
